# Mirror Visual Feedback Prior to Robot-Assisted Training Facilitates Rehabilitation After Stroke: A Randomized Controlled Study

**DOI:** 10.3389/fneur.2021.683703

**Published:** 2021-07-08

**Authors:** Jifeng Rong, Li Ding, Li Xiong, Wen Zhang, Weining Wang, Meikui Deng, Yana Wang, Zhen Chen, Jie Jia

**Affiliations:** ^1^The Center of Rehabilitation Therapy, The First Rehabilitation Hospital of Shanghai, Shanghai, China; ^2^The Department of Rehabilitation Medicine, Huashan Hospital, Fudan University, Shanghai, China; ^3^The Neurorehabilitation Centre, The First Rehabilitation Hospital of Shanghai, Shanghai, China; ^4^The National Clinical Research Center for Aging and Medicine, Huashan Hospital, Fudan University, Shanghai, China

**Keywords:** embodiment, mirror visual feedback, robot-assisted training, stroke, upper limb

## Abstract

**Purpose:** Robot-assisted training has been widely used in neurorehabilitation, but its effect on facilitating recovery after stroke remains controversial. One possible reason might be lacking consideration of the role of embodiment in robotic systems. Mirror visual feedback is an ideal method to approach embodiment. Thus, we hypothesized that mirror visual feedback priming with subsequent robot-assisted training might provide additional treatment benefits in rehabilitation.

**Method:** This is a prospective, assessor-blinded, randomized, controlled study. Forty subacute stroke patients were randomly assigned into an experimental group (*N* = 20) or a control group (*N* = 20). They received either mirror visual feedback or sham-mirror visual feedback prior to robot-assisted training for 1.5 h/day, 5 days/week for 4 weeks. Before and after intervention, the Fugl-Meyer Assessment Upper Limb subscale, the Functional Independence Measure, the modified Barthel Index, and grip strength were measured. Scores of four specified games were recorded pre and post one-time mirror visual feedback priming before intervention in the experimental group.

**Results:** All measurements improved significantly in both groups following interventions. Moreover, the Fugl-Meyer Assessment Upper Limb subscale, self-care subscale of the Functional Independence Measure, and the grip strength were improved significantly in the experimental group after a 4-week intervention, compared with the control group. Significantly higher scores of two games were revealed after one-time priming.

**Conclusions:** Mirror visual feedback prior to robot-assisted training could prompt motor recovery, increase ability of self-care, and potentially enhance grip strength in stroke patients, compared to control treatment. Moreover, mirror visual feedback priming might have the capability to improve the patient's performance and engagement during robot-assisted training, which could prompt the design and development of robotic systems.

**Clinical Trial Registration:** www.ClinicalTrials.gov, identifier: ChiCTR1900023356.

## Introduction

Approximately 75% of stroke patients suffer from upper limb motor impairments, which are challenging and complex to restore (1). Since the first employment in clinical study (2), robot-assisted training (RT), as an intensive and task-specific intervention, has been increasingly used in neurorehabilitation and numerous studies have reported its potential to facilitate upper limb rehabilitation (3–7). Recent studies suggested that repetitions of movement and patient engagement are determinants in neural plasticity, which is of great importance for prompting rehabilitation (8, 9). With engagement increasing, patient–robot interaction, which plays a critical role in robotic systems, could be enhanced (10). Moreover, patients with strong motivation could pay more attention and could actively attempt to accomplish physical exercise during RT. Therefore, approaches and strategies, such as virtual reality (VR) and assist-as-needed, have been proposed to increase patient participation and motivation in RT (11). However, to the best of our knowledge, there are few methods and research focusing on reinforcing patient engagement or attention from the perspective of embodiment prior to RT.

Embodiment, also called bodily self-consciousness, is a kind of experience, which comprise four basic components, namely, body ownership, location, agency, and deafference (12, 13). Illusions stemming from rubber hand, mirror visual feedback (MVF), and VR have been suggested to evaluate aspects of embodiment (14–17). Research on stroke rehabilitation demonstrate that the experience of embodiment during these visual stimulations, like MVF, has the potential to alter the patient's sensorimotor activity and contribute to motor recovery (18–20). Studies reported that MVF priming had an instant effect on neural modulation and a long-term effect on neural plasticity, which was recognized as the underlying therapeutic mechanism (20–23). Moreover, patients with hemiparalysis after stroke may obtain more benefits on motor function restoration through the substitution of paralyzed limbs with visual inputs of the active side controlled by the patients themselves (24, 25). Wainer et al. reported that embodiment could affect patients' engagement and presented a positive correlation between the embodiment perception and the effectiveness of RT (26). Thus, we inferred that lacking education or perception of embodiment might limit the benefits achieved from RT in patients with stroke. Additionally, our previous studies demonstrated that the embodiment deriving from MVF could enhance the patient's attention to affected upper limb, which might strengthen the control of paralyzed limbs and hinder the development of learned non-use after stroke (18, 27). Therefore, we hypothesized that using MVF as a priming technique to promote the patient's attention to affected limbs and train the embodiment perception prior to RT might facilitate the rehabilitation process after stroke.

In the present study, an arm rehabilitation robot and a customized camera-based MVF (camMVF) (18, 27) were employed to provide robot-assisted upper limb training and MVF for patients after stroke. A randomized controlled study was designed to investigate the potential effects of MVF prior to RT on motor function, daily activities, grip strength, and gamified training performance.

## Methods

This was a 4-week prospective, assessor-blinded, randomized, controlled trial. The Institutional Review Boards of Huashan Hospital, Fudan University approved the study (KY2017-230), and it was registered on the Chinese clinical trial registry (registration number: ChiCTR1900023356). All the subjects were inpatients, who were recruited from the First Rehabilitation Hospital of Shanghai, as a branch center of Huashan Hospital, from January 2019 to December 2020. All patients were informed of the goal and protocol of the study, and signed informed consent forms prior to the participation. The demographic characteristics of patients and all the measurements were conducted by an independent therapist pre- and post-intervention. The allocation sequence was based on a computer-generated random number table. Sealed and numbered envelopes were created to allocate patients. The randomization program and all the assignments were conducted by an independent researcher.

**Figure 1:**
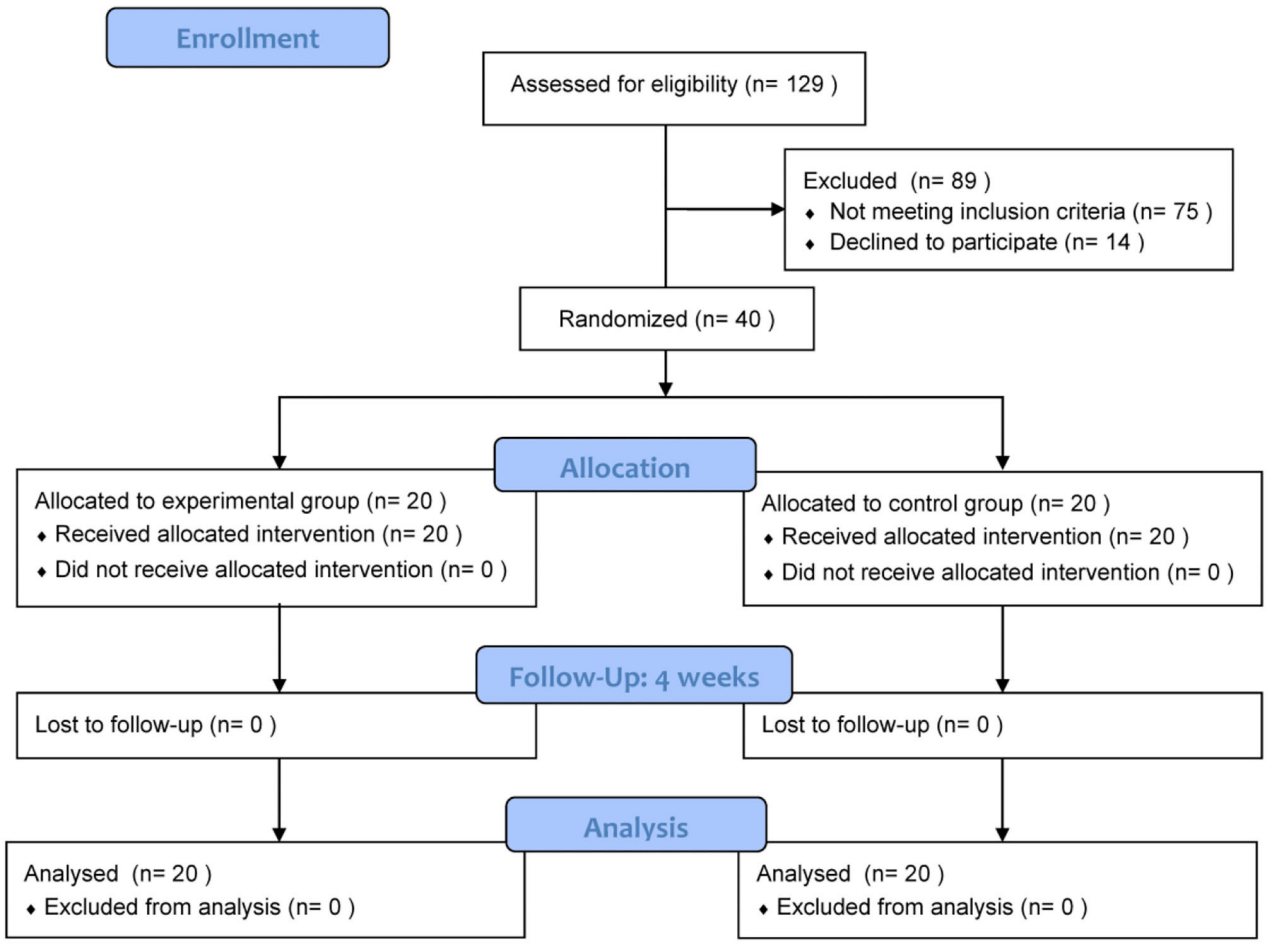
Flow chart of patients through this study.

After baseline assessment, eligible patients were randomly assigned into an experimental group or a control group (see Figure 1). Patients in the experimental group received MVF prior to robot-assisted training (MRT group), while the control group received sham-MVF with subsequent robot-assisted training (RT group). The inclusion criteria were as follows: (1) diagnosed as unilateral stroke for the first time, (2) within 1 month to 6 months after stroke onset, and (3) age between 18 and 80 years. The exclusion criteria included severe cognitive impairment (MMSE ≤ 23), and severe pain or sensory impairment.

In order to detect differences in the effects of *time* × *group* interactions on the primary outcome (the Fugl-Meyer Assessment Upper Limb, FMA-UL), an estimation of sample size was carried out. The effect size from 0.1 to 0.5 is expected, based on previous studies in the field (4, 27, 28). Thus, a total of 40 participants (20 per arm) was estimated as an optimal sample size for this study according to the stepped rules of thumb (29).

### Intervention

Patients were separated into two groups and received corresponding treatment for 1.5 h per day, 5 days per week, for 4 weeks. All the treatments were conducted by experienced physiotherapists.

### camMVF Prior to RT

**Figure 2:**
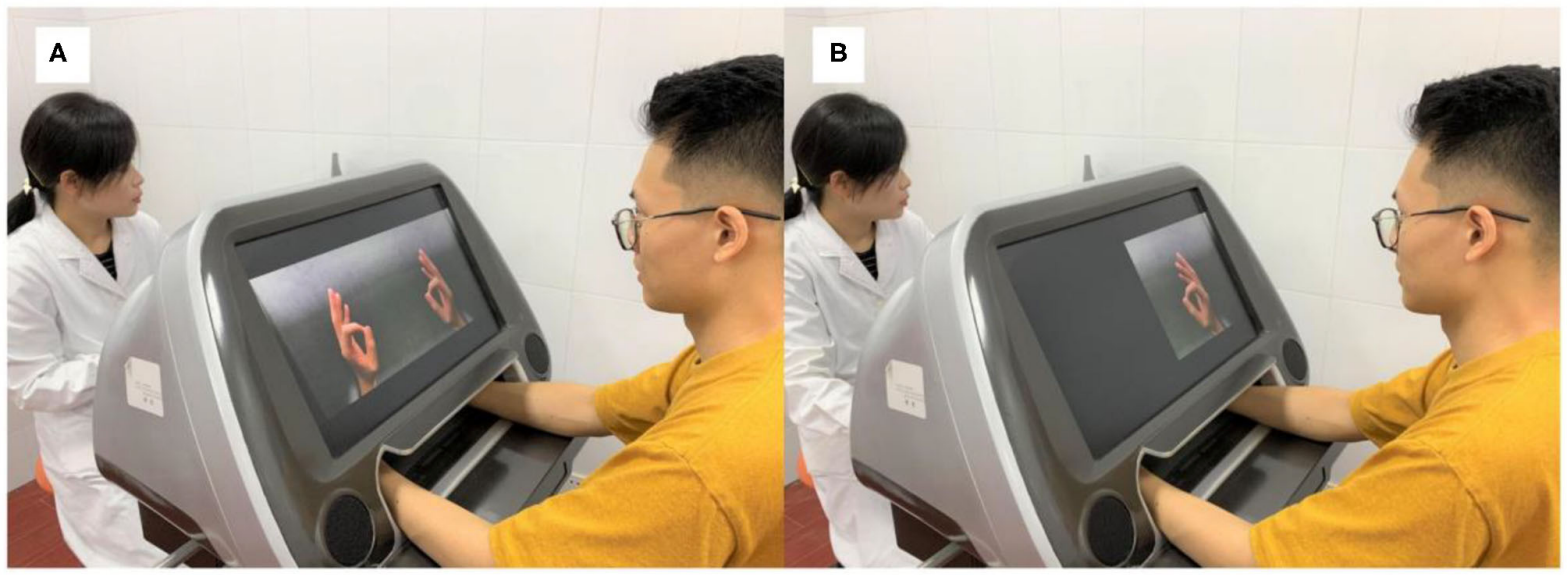
The camera-based mirror visual feedback setup used in the study. **(A)** Providing mirror visual feedback. **(B)** Sham-mirror visual feedback.

In this study, patients in the experimental group received MVF priming with subsequent RT. A customized camMVF was employed to provide MVF (Figure 2A). The MVF priming aimed to enhance the patient's attention to the affected side and strengthen the embodiment perception (18, 27). During MVF, the pictures of unaffected arms and its mirror image were shown on a screen in front of the patients. Two types of training were contained in the computerized device: (1) motor training, emphasizing motor exercise of finger, hand, wrist, and forearm; (2) task training, including object-based reaching, grasping, and placing. The MVF priming lasted for 0.5 h. The instructions were “Keep your eyes on the screen, pay attention to the reflection of the hand, and try to imagine it is your affected one” and “During training, move both your hands synchronously. But if it is hard, you should rest the affected side.”

**Figure 3:**
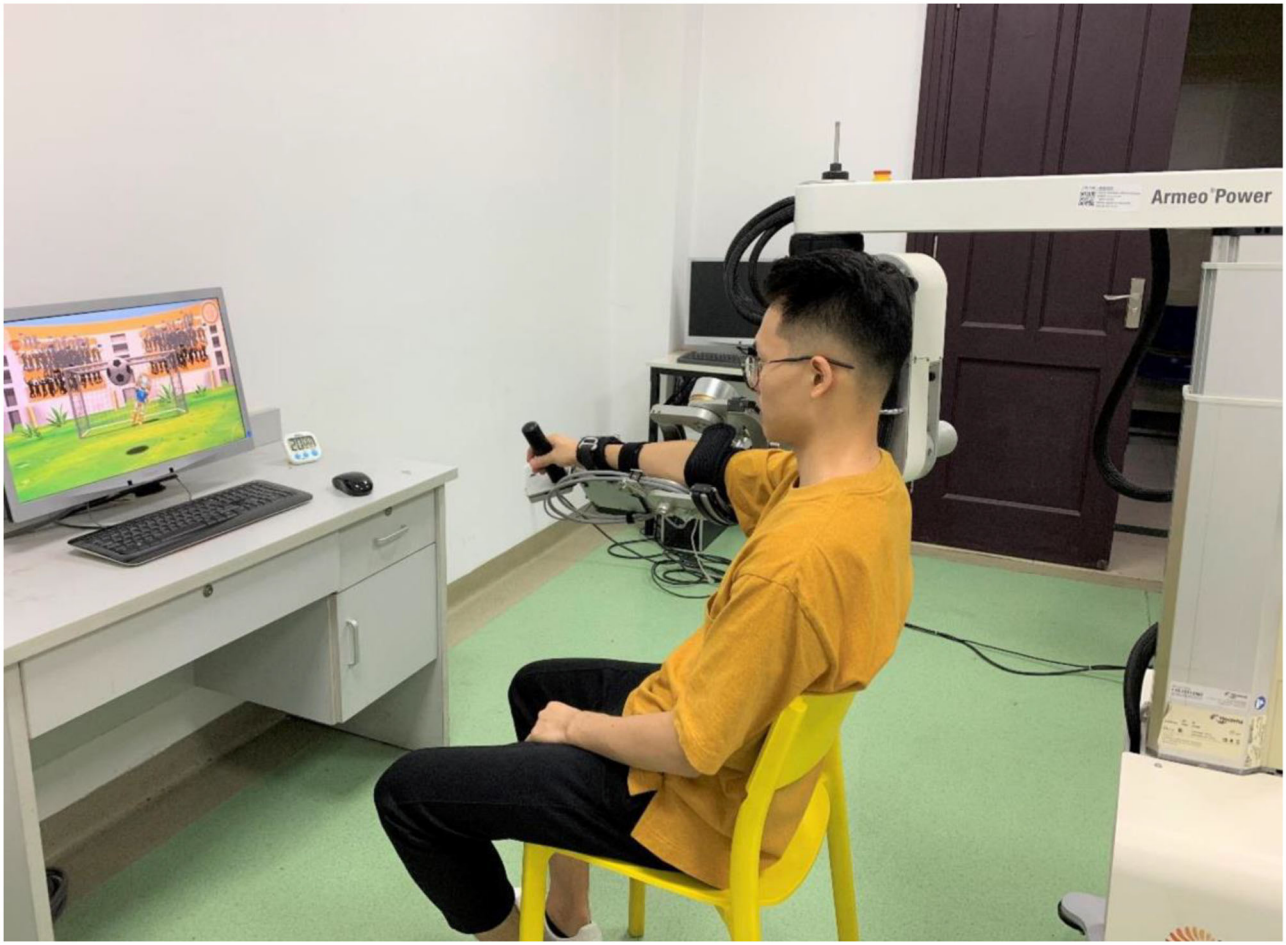
The robotic system used in the study for upper limb rehabilitation.

Subsequently, 1 h RT was provided, using Armeo Power (Figure 3) (Hocoma, Volketswil, Kanton Zürich, Switzerland). Armeo is an assistive exoskeleton, which can support the patient's arm weight and provide a feeling of fluctuation (30). The device provides arm weight support and custom software, which excels in motivating patients *via* various engaging games and functional training that simulate daily activities. Four games were chosen to enable patients to relearn upper limb motor abilities and to train functional exercises, namely, balloon collection (G1), goalkeeper (G2), water collection (G3), and monster rescue (G4). These four games focused on motor training of the upper limb, forearm, and wrist, and hand grasp, respectively (see Supplementary Material for details). Each game was played for 5 min (including a 2-min rest period), repeating three times.

### Control Treatment

Patients in the control group received 1.5 h dosage-equivalent (intensity and duration) exercises as the experimental group, which comprised 0.5 h sham-MVF (31) training (Figure 2B) prior to 1 h RT. During sham-MVF, the reflection of the affected side will be shielded to restrain the development of mirror illusion, which related to subjective embodiment experience. However, the same instructions were provided and patients were still required to attempt symmetrical movement. After sham-MVF training, four gamified trainings were provided, and the protocol was in accordance with the experimental group.

### Outcome Measures

The FMA-UL, which was widely used in studies on neurorehabilitation, was employed as one of the primary measurements in our present study to evaluate the motor impairment and recovery of upper limb (21). The FMA-UL (maximum: 66) applied a three-point ordinal scale from 0 to 2 to assess upper limb function, in which “0” represented “cannot perform,” “1” represented “can perform partially,” and “2” represented “can perform fully.” A study indicated that FMA-UL ≤ 34 indicated severe to moderate motor impairment and FMA-UL ≥ 35 represented moderate–mild (32). In order to evaluate hand function recovery and further investigate the effect of treatments, the score of wrist and hand of the FMA (FMA-WH, maximum: 24) was also employed.

The Functional Independence Measure (FIM) (33) and the modified Barthel Index (MBI) (34) were applied to measure improvements in activities of daily living (ADLs), and the FIM was regarded as the other primary measurement. The FIM (maximum: 126) measured independent functions and is composed of six subscales, namely, self-care, sphincter control, transfers, locomotion, communication, and social cognition, which were analyzed separately for specific investigation. The MBI (maximum: 100) was used to measure the patient's performance in 10 aspects of ADLs, with higher scores indicating better performance. In order to investigate the effect of robotic training on muscle power of hand, grip strength test was applied, using a Jamar Hydraulic Hand Dynamometer.

Moreover, we evaluated and compared the scores of the four prescript games pre and post one-time camMVF-based training before the first intervention for patients in the experimental group, to investigate the instant influence of MVF priming on gamified training performance. After the baseline assessment, 20 eligible patients assigned into the experimental group participated in this measurement. Firstly, patients were required to conduct the gamified testing of the RT without priming. After a 6-h interval, they received 0.5 h MVF priming and subsequently completed the testing. Each game included three sessions (lasting for 3 min), and the average scores for each game were calculated. All the measurements were conducted before the first intervention.

### Statistical Analysis

Patients' characteristics were compared between two groups using Fisher's exact test (gender, type, and side of stroke), one-way ANOVA (age and months after stroke), and Mann–Whitney *U*-tests (Brunnstrom stages). The differences of the outcomes, including the FMA-UL, the FMA-WH, the FIM, the subscales of the FIM, the MBI, and grip strength, between groups were analyzed using two-way repeated measures ANOVAs, taking group as between factor and time as within factor. If any significant *time* × *group* interaction was obtained, *post-hoc* analysis was performed with Bonferroni correction. Paired *t*-test was employed to compare the difference on the scores of four selected games in the experimental group. Data analysis was conducted using SPSS version 24.0. The normality of data was evaluated by Shapiro–Wilk's test and the homogeneity of variances was checked by Levene's test. Results were presented as mean with standard deviations (SD). The significance level was set at *p* < 0.05 with a two-sided test.

## Results

A total of 40 patients (experimental group, *N* = 20; control group, *N* = 20) were recruited from the First Rehabilitation Hospital of Shanghai. No patients dropped out from this study, and no adverse events were reported. Table 1 presents the demographic characteristics of patients in both groups and no significant differences between the two groups were revealed.

**Table 1:** Demographic characteristics of patients.

**Characteristics**	**MRT group (*N* = 20)**	**RT group (*N* = 20)**	***p***
Age (years), mean (SD)	56.25 (12.29)	62.30 (13.10)	0.140
Months after stroke onset, mean (SD)	3.65 (1.53)	3.85 (1.79)	0.706
Gender, *n*			0.451
Male	17	14	
Female	3	6	
Type of stroke, *n*			0.301
Ischemia	12	16	
Hemorrhage	8	4	
Side of paralysis, *n*			0.751
Right	10	8	
Left	10	12	
Brunnstrom stages, mean (SD)			
Proximal	3.20 (1.11)	3.40 (1.14)	0.640
Distal	3.30 (1.17)	3.75 (1.25)	0.242

*MRT, experimental group that received robot-assisted training combining camera-based mirror visual feedback; RT, control treatment group*.

### Motor Impairment

The statistical results of the FMA-UL and the FMA-WH scores are shown in Table 2. Significant *time* × *group* interaction was only found on the FMA-UL [*F*_(1,38)_ = 25.532, *p* < 0.001]. *Post-hoc* analysis indicated that the FMA-UL scores of both groups were significantly increased after a 4-week intervention (*p* < 0.001 for both groups; ΔFMA-UL_post−pre_: MRT: 15.60, RT: 9.50; ΔFMA-WH_post−pre_: MRT: 4.25, RT: 3.35). Moreover, the scores were comparable between the two groups before intervention (*p* = 0.685) and the scores in the experimental group were significantly higher than the control group after a 4-week intervention (*p* = 0.048). This finding suggested that patients in the experimental group achieved more restoration of motor function than those in the control group. There was no significant interaction on the FMA-WH [*F*_(1,38)_ = 2.437, *p* = 0.127]. A significant main effect of time (*p* < 0.001) was revealed, suggesting that the FMA-WH scores were significantly improved after intervention. However, no significant main effect of the group was found [*F*_(1,38)_ = 2.077, *p* = 0.158].

**Table 2:** Descriptive and inferential statistics for motor impairment, daily function, and grip strength.

	**MG (*****N*** **=** **20)**				**CG (*****N*** **=** **20)**				**ANOVA**	
	**Pretest**		**4 weeks after**		**Pretest**		**4 weeks after**		***F***	***p***
**FMA**	**Mean (SD)**	**95% CI**	**Mean (SD)**	**95% CI**	**Mean (SD)**	**95% CI**	**Mean (SD)**	**95% CI**		
FMA-UL	31.75 (13.12)	25.61–37.89	47.35 (11.81)	41.82–52.88	30.10 (12.43)	24.28–35.92	39.60 (12.21)	33.88–45.32	25.532	<0.001^*^
FMA-WH	12.25 (5.91)	9.48–15.02	16.50 (5.21)	14.06–18.94	10.50 (4.15)	8.56–12.44	13.85 (4.16)	11.90–15.80	2.437	0.127
**MBI**	58.50 (22.66)	47.90–69.10	71.75 (21.73)	61.58–81.92	52.00 (13.71)	45.58–58.42	62.90 (13.33)	56.66–69.14	2.599	0.115
**FIM**										
Total	65.56 (4.17)	63.70–67.60	80.95 (4.65)	78.77–83.13	63.75 (5.30)	61.27–66.23	76.35 (5.41)	73.82–78.88	3.061	0.088
Self-care	16.55 (2.61)	15.33–17.77	25.45 (2.37)	24.34–26.56	15.65 (2.06)	14.69–16.61	22.60 (2.68)	21.34–23.86	4.505	0.040^*^
Sphincter control	12.45 (0.69)	12.13–12.77	13.50 (0.51)	13.26–13.74	12.60 (0.82)	12.22–12.98	13.55 (0.51)	13.31–13.79	0.087	0.770
Transfers	8.70 (0.92)	8.27–9.13	9.70 (0.86)	9.30–10.10	8.70 (1.08)	8.19–9.21	10.15 (1.57)	9.42–10.88	2.083	0.157
Locomotion	6.00 (1.21)	5.43–6.57	6.95 (1.43)	6.28–7.62	5.75 (1.12)	5.23–6.27	6.35 (1.57)	5.62–7.08	0.975	0.330
Communication	11.40 (1.10)	10.89–11.91	12.80 (0.95)	12.35–13.25	11.05 (1.28)	10.45–11.65	12.30 (0.80)	11.93–12.68	0.121	0.730
Social cognition ability	10.55 (1.90)	9.66–11.44	12.55 (2.04)	11.60–13.50	10.00 (1.65)	9.23–10.77	11.40 (1.57)	10.67–12.13	2.178	0.148
**Grip strength**	2.48 (0.78)	2.11–2.85	3.28 (0.65)	2.97–3.59	1.71 (1.38)	1.06–2.35	2.22 (1.03)	1.74–2.70	4.179	0.048^*^

*MRT, experimental group that received robot-assisted training combining camera-based mirror visual feedback; RT, control treatment group; ANOVA, analysis of variance; FMA-UL, Fugl-Meyer Assessment Upper Limb subscale; FMA-WH, scores of wrist and hand of FMA-UL; MBI, modified Barthel Index; FIM, Functional Independence Measure, CI, confidence interval*.

* *p < 0.05*.

### Daily Function

For daily function, no significant *time* × *group* interactions were found on the FIM [*F*_(1,38)_ = 3.061, *p* = 0.088] or the MBI [*F*_(1,38)_ = 2.599, *p* = 0.115] (see Table 2). However, a significant main effect of time and group (*p* < 0.001 and *p* = 0.021, respectively) for the FIM was obtained, which indicated that the FIM scores were significantly improved after intervention in both groups, respectively (*p* < 0.001 for both groups; ΔMBI_post−pre_: MRT: 13.25, RT: 10.90; ΔFIM_post−pre_: MRT: 15.39, RT: 12.60). Besides, a significant interaction was found on the self-care subscale of FIM [*F*_(1,38)_ = 4.505, *p* = 0.040]. Further analysis showed that the scores of self-care subscale were significantly increased in MRT after intervention, comparing with RT (pre: MG: 16.55 ± 2.61, CG: 15.65 ± 2.06, *p* = 0.233, post: MG: 25.45 ± 2.37, CG: 22.60 ± 2.68, *p* = 0.001). Only a significant main effect of time (*p* < 0.001) was found for the MBI. The scores of FIM, subscales of FIM, and MBI were comparable between the two groups before intervention (all *p* > 0.05).

### Grip Strength

ANOVA on grip strength demonstrated a significant *time* × *group* interaction [*F*_(1,38)_ = 4.179, *p* = 0.048, Table 2]. Further analyses showed that both groups demonstrated a significant increase after 4 weeks of intervention (*p* < 0.001 for both groups; Δ_post−pre_: MRT: 0.80, RT: 0.51). However, further analysis indicated that the grip strength was not comparable between the two groups before intervention (*p* = 0.035). Thus, ANCOVA was applied on this measure, which demonstrated a significant difference between MRT and RT after intervention [estimated marginal means, MRT: 3.00, 95% CI: 2.86–3.15; RT: 2.50, 95% CI: 2.35–2.64, *F*_(1, 37)_ = 23.449, *p* < 0.001]. This suggested that patients who received the intervention of RT combined with camMVF might achieve more improvements in grip strength.

### Gamified Training Performance

**Figure 4:**
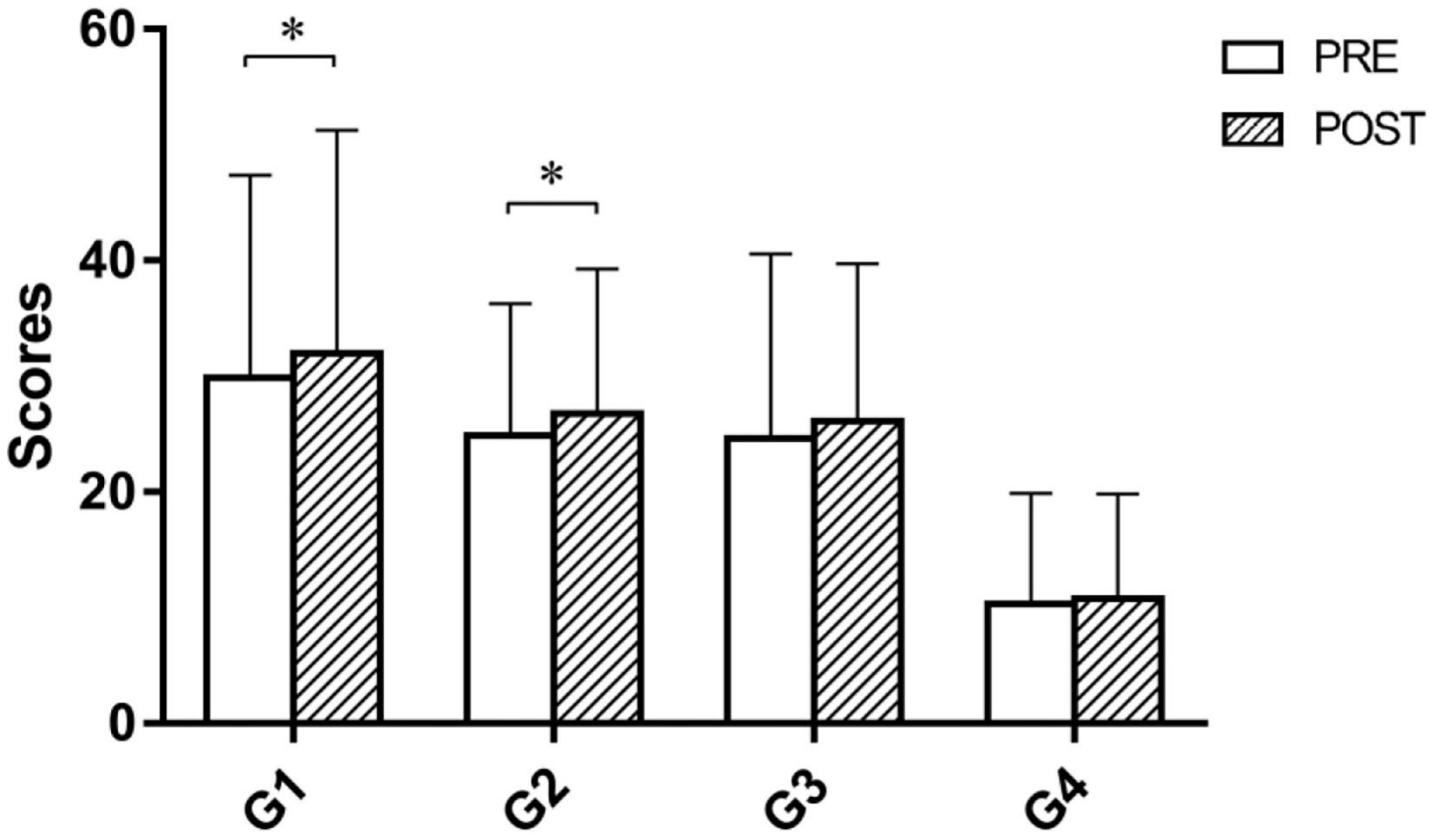
Scores of four prescribed games pre and post priming before intervention in the MRT group. The scores of G1 and G2 were significantly improved after priming. G1, balloon collection; G2, goalkeeper; G3, water collection; G4, monster rescue. Error bars represent the standard deviation of the mean.*p < 0.05.

The results of the paired *t-*test on the scores of four games are reported in Figure 4. After one-time camMVF-based training, the scores of G1 and G2 significantly increased. However, no significant differences were obtained for G3 and G4.

## Discussion

As a study exploring the effect of MVF prior to RT on facilitating rehabilitation after stroke, our study firstly provides additional evidence from the perspective of embodiment that the integration of these two approaches is a superior combination in reducing upper limb motor impairment and improving the ability of self-care in patients with stroke. This combination also presented a potential to enhance the patient's grip strength. Moreover, our study suggests that mirror visual priming could have the capability to improve the patient's performance and engagement during RT, which might prompt the development of robotic systems in the field of rehabilitation.

RT is an intensive and task-specific intervention, which has been widely employed in neurorehabilitation, especially for motor dysfunction of upper limbs (3–7). In line with previous studies (30, 35, 36), our study revealed decreased motor impairment of upper limb, improved daily function, and increased grip strength in both groups, where patients received Armeo Power training. Studies have indicated that arm weight supported training, which allowed active repetitive motor training, could increase motor function of stroke patients *via* enriching sensorimotor input (36, 37). This might be the reason why there were improvements for patients with stroke after the intervention of RT in our study. Besides, the gamified functional training of this device might motivate patients and contribute to motor recovery and improvement in ADLs.

Some researchers reported that RT alone had limited effects on stroke recovery (38, 39). In order to facilitate rehabilitation, recent robotic systems provided intensive, repetitive, task-oriented training with visual feedback (38, 39). However, these studies, as well as clinical practice, take on little consideration of the patient's motivation during RT. Although gamified training emphasizing entertainment was employed in some robotic systems, few strategies were proposed to enhance the embodiment perception. Embodiment relates to the sense of self, of which the degree varies in populations (12). It can influence the patient–robot interaction during training tasks, and there is a positive correlation between the subjective embodiment perception and effectiveness of treatment (26). Thus, we speculated that the patient's variability in embodiment might result in inconsistent findings. MVF prompts multisensory integration in stroke patients and generates attention to affected limbs; moreover, it has the ability to make patients embody the reflected limbs, which might be used as a training method to reduce the variability in embodiment (15, 16). In the present study, the camMVF was employed to prime patients before RT, which aimed to make patients experience embodiment and motivate them for subsequent robotic training. Moreover, the sham-MVF acted as a motion observation approach, which presented direct visual stimulation. The results of the study demonstrated that patients who received RT combined with MVF achieved more improvements in motor recovery, ability of self-care, and even grip strength, comparing to those without mirror visual priming, which further confirmed the superiority of this strategy. Our previous study suggested that subacute stroke patients with severe-moderate motor impairment benefited more from MVF (27). In the present study, patients with 1 to 6 months after stroke onset were recruited and demonstrated moderate motor impairment (mean FMA-UL ≤ 34) (32). These patient characteristics might also contribute to the improvements in MRT. Although the grip strength was incomparable at baseline in the present study, a limited relationship between the grip strength and the motor recovery was proposed for the spasticity phase and the intention of the FMA (40). However, this might still have potential influence on the results and future studies should consider the impact of grip strength, which we recognized as a study limitation.

As a visual input stimulation approach, MVF can induce a sense of mirror illusion, which stems from a misperception of ownership (16, 41). In our supplemental investigation, higher scores for robotic games were observed after MVF priming, which might indicate a better performance of robotic training. One possible interpretation is that the strengthened sense of body ownership could generate the patient's attention to the affected side, which might increase motor control of paralyzed limbs and contribute to better training performance. Although very limited evidence is presented, we speculate that patients might generalize the experience of embodiment during MVF to the interaction of robots, which can indirectly enhance the immersion and operability of gamified training. This finding also suggests the extended use of MVF in various treatments involving interactions between human and substitutions to facilitate recovery.

Studies showed that MVF has an instant effect on neural modulation, including activation of the sensorimotor cortex and normalization of interhemispheric inhibition (19, 20, 42). Thus, MVF can be used to pre-activate the motor system and intercortical circuitries (43, 44), which might facilitate the effects of subsequent RT. It also presents an effect on neural plasticity, which leads to an alteration of activation patterns of the motor cortex and efficacy of the brain network (18, 22, 23, 25). These might also be one of the interpretations for the improvements of patients in motor recovery and self-care in the present study.

### Study Limitations

There were several limitations. First, subjective embodiment perception was not measured in our study. Questionnaires on embodiment could be employed in future exploration. Secondly, although our number of participants was estimated in the study, a small sample size may still hinder the power of statistical analyses. Third, the ANCOVA was applied on grip strength, but further investigations are still needed for incomparable grip strength data at the baseline. Moreover, comparisons among trials assessing the efficacy of MRT, MVF, and RT alone should be made in future studies; meanwhile, electrophysiological or functional imaging approaches should be considered for further investigation of neural reorganization as underlying mechanisms of this approach.

## Conclusion

To our knowledge, this study is the first to investigate the effect of RT combining customized camera-based MVF on upper limb rehabilitation from the perspective of embodiment. Our results revealed that mirror visual priming with subsequent RT was a superior combination over the control treatment in reducing upper limb motor impairment and improving ability of self-care in patients with stroke. Also, this combination presented a potential to enhance the patient's grip strength.

## Data Availability Statement

The raw data supporting the conclusions of this article will be made available by the authors, without undue reservation.

## Ethics Statement

The studies involving human participants were reviewed and approved by the Institutional Review Boards of Huashan Hospital, Fudan University. The patients/participants provided their written informed consent to participate in this study. Written informed consent was obtained from the individual(s) for the publication of any potentially identifiable images or data included in this article.

## Author Contributions

JJ, ZC, JR, and LD: study concept and design. LX, WZ, and WW: acquisition of data. MD and YW: analysis and interpretation. JR and LD: drafting of manuscript. JJ and ZC: critical revision of manuscript and study supervision. All authors contributed to the article and approved the submitted version.

## Conflict of Interest

The authors declare that the research was conducted in the absence of any commercial or financial relationships that could be construed as a potential conflict of interest.
