# Supplementary material for: Mirror Visual Feedback Prior to Robot-Assisted Training Facilitates Rehabilitation After Stroke: A Randomized Controlled Study
Source: Front Neurol. 2021 Jul 8;12:683703. doi: 10.3389/fneur.2021.683703 (PMC8297738; doi:10.3389/fneur.2021.683703)
Supplement: Supplementary file 1 [file Data_Sheet_1.PDF]

Introduction to Games of Armeo

Armeo software contains a large number of game sports exercises, all exercises appear in a virtual reality training environment, which can motivate the user, but also challenging, giving feedback while clearly demonstrating functional tasks. These motivating exercises that require users to perform independently mainly cover the following functions: Grasp and relax, Pronation and supination, Wrist flexion and extension, Reach and grab. The system can detect extremely small movements and functions, and assist users in reaching and grasping with higher intensity.

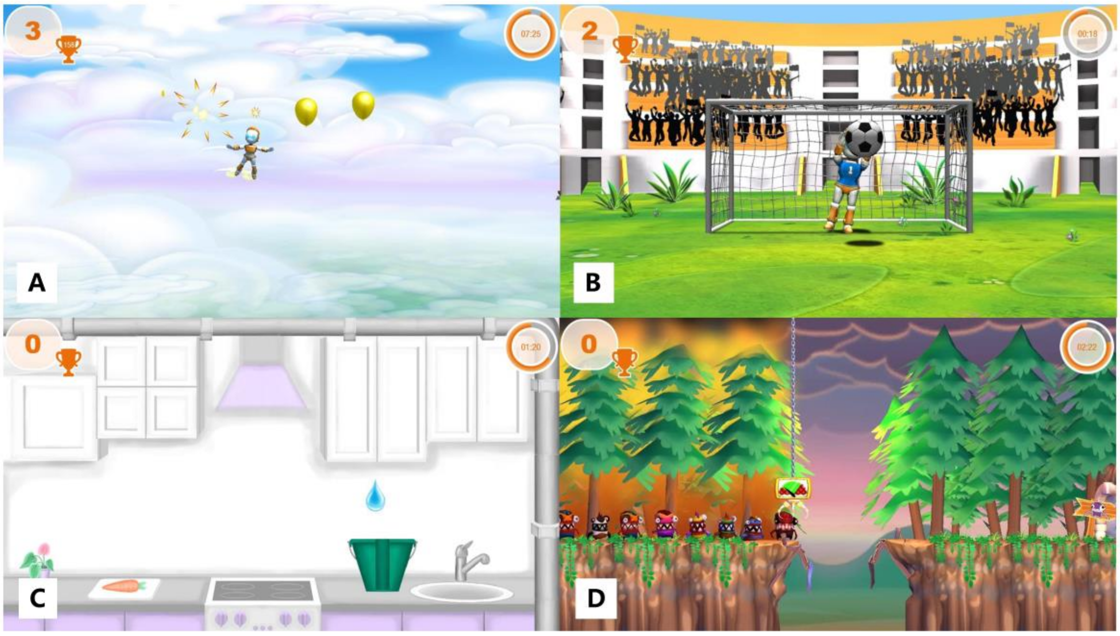

- **balloon collection (A)**

Content and process:

This is a scenario simulation training that can train multiple joints. The patient's affected upper limb is transformed into a little person wearing a helmet, controlling the little person's flight position, and using a small nail in the sky to pierce the aircraft from the aircraft. Balloons falling apart. First, you have to control the little man to reach the position of the aircraft, and then the balloons will spread out, then control the little man to touch the balloons until all the balloons are punctured, the first level task is completed, and then there will be The aircraft with more balloons appears, repeat the previous steps to destroy all balloons.

Difficulty adjustment and score:

the game set the time, as the progress of the game, more balloons will appear, there are bombs mixed in the balloons, the more balloons burst within the specified time, the higher the score, but if accidentally touched the bomb, the score will be reduced.

Functions of training:

mainly exercise the extension, joint and separation of the upper limbs, mainly involving the flexion and extension of the shoulder joint, adduction and abduction, elbow flexion and extension of these degrees of freedom functions.

- **goalkeeper (B)**

Content and process:

This is a scenario simulation training for single joint training. From the patient's interest point of view, it can improve the patient's motivation for training. The patient's forearm controls the

inclination angle of the goalkeeper's body, and throws off the football kicked on the left and right sides of the door frame. The position of the goalkeeper's foot is always in the middle of the bottom of the door frame and only changes the body angle.

Difficulty adjustment and scoring:

More footballs will come oncoming, and the goalkeeper needs to react quickly over the time.

In the process of football launch, there are sneakers. If you accidentally pounce on the sneakers, the score will be reduced. The higher the difficulty, the faster the football can enter the goal and the more shoes are mixed.

Functions of training:

Mainly exercise forearm pronation and supination. Pronation controls the goalkeeper to lean left to catch the ball, while supination controls the goalkeeper to catch the ball right. In the training process, the shoulder joint will keep in the proper position to reduce the compensation of other joints.

- **water collection (C)**

Content and process:

This is an ADL simulation training for single joints. In the kitchen scene, you can use your wrist to control the horizontal movement of the water cup and catch the water drops falling from it. Different water drops will fall at different positions.

Difficulty adjustment and scoring:

The more drops you receive, the higher the score. The more difficult the drops are, the faster they will fall and the smaller they will become. This requires precise control of the cup to a specific position.

Training feature:

This game uses single joint movement for training. The patient uses wrist flexion and extension to control the horizontal position of the cup, and the shoulder and elbow are fixed in the appropriate neutral position to reduce compensation. The greater the Angle of flexion and extension of the elbow, the farther the glass will move.

- **monster rescue (D)**

Content and flow:

This is a situational game that trains multiple joints and focuses more on hand grip and release. There is a big river between the two cliffs. There are many small monsters queuing up to cross to the other side of the cliff on the left side, and the fire is about to burn to the small monster behind. The upper limb of the patient is the pickup device of the rescue machine, and the small monster on one side of the cliff crosses the abyss and escapes from the other side of the cliff. Patients need to control their upper limbs to reach the small monster and grasp it with their hands before reaching the opposite bank. Only when they reach the bank safely can they relax and put down the small monster. Repeat the previous action to save the other monsters in line.

Difficulty and score:

The more monsters saved, the higher the score. As the game progresses, the height of the cliffs on both sides will change, which means that patients need to control the pickup clips to carry out more activities. The fire behind the little monsters will spread slowly, and the score will be reduced if the little monsters are burned.

Functional training:

Mainly for upper limb activities, especially for hand grip and relaxation. In the process of reaching

and grasping little monsters, hand functions should be coordinated to grasp and relax at the appropriate time. The functional requirements for the upper limbs are higher. This idea is that you want the patients to make large numbers of movements, supported by the robot early after stroke. we want them to make hundreds if not thousands of exploratory movements. it is a very massive, exploratory, playful motivating environment, and we are having the patients cover about one kilometer to two kilometers per session, that is so exciting to the patients, so they don' t realize that they are doing as I said kilometers of arm movement every single day.
